# Responsive robotic prey reveal how predators adapt to predictability in escape tactics

**DOI:** 10.1073/pnas.2117858119

**Published:** 2022-06-03

**Authors:** Andrew W. Szopa-Comley, Christos C. Ioannou

**Affiliations:** ^a^School of Biological Sciences, Life Sciences Building, University of Bristol, Bristol BS8 1TQ, United Kingdom

**Keywords:** predation, protean behavior, unpredictable behavior, blue acara, *Andinoacara pulcher*

## Abstract

To increase their chances of survival, prey often behave unpredictably when escaping from predators. However, the response of predators to, and hence the effectiveness of, such tactics is unknown. We programmed interactive prey to flee from an approaching fish predator (the blue acara, *Andinoacara pulcher*) using real-time computer vision and two-wheeled robots that controlled the prey’s movements via magnets. This allowed us to manipulate the prey’s initial escape direction and how predictable it was between successive trials with the same individual predator. When repeatedly exposed to predictable prey, the predators adjusted their behavior before the prey even began to escape: prey programmed to escape directly away were approached more rapidly than prey escaping at an acute angle. These faster approach speeds compensated for a longer time needed to capture such prey during the subsequent pursuit phase. By contrast, when attacking unpredictable prey, the predators adopted intermediate approach speeds and were not sensitive to the prey’s escape angle but instead showed greater acceleration during the pursuit. Collectively, these behavioral responses resulted in the prey’s predictability having no net effect on the time taken to capture prey, suggesting that unpredictable escape behavior may be advantageous to prey in fewer circumstances than originally thought. Rather than minimizing capture times, the predators in our study appear to instead adjust their behavior to maintain an adequate level of performance during prey capture.

## Significance

A widespread strategy used by prey animals, seen in insects, mammals, amphibians, crustaceans, fish, and reptiles, is to vary the direction in which they escape when attacked by a predator. This unpredictability is thought to benefit prey by inhibiting predators from predicting the prey’s escape trajectory, but experimental evidence is lacking. Using fish predators repeatedly tested with interactive, robot-controlled prey escaping in the same (predictable) or in random (unpredictable) directions, we find no clear benefit to prey of escaping unpredictably, driven by behavioral counteradaptation by the predators. The benefit of unpredictable escape behavior may depend on whether predators are able to counteract prey escape tactics by flexibly modifying their behavior, or unpredictability may instead be explained biomechanical or sensory constraints.

Rapid evasive responses are a vital tool prey use to minimize capture by predators (1, 2). Despite their ubiquity, it can be challenging to demonstrate the benefit of escape strategies, due to the difficulties involved in designing studies which integrate realistic predation with manipulation of prey behavior that experimentally controls for confounding effects. Studying the behavior of real predators is crucial when attempting to demonstrate the adaptive value of prey adaptations, especially when these are dependent on features of predator cognition (3–5). This applies particularly to unpredictable escape behavior by prey, which is thought to enhance prey survival by compromising the ability of predators to anticipate the movement of their target (6). Although unpredictable escape tactics are widespread taxonomically (7, 8), we know little about how real predators respond to unpredictability in prey escape strategies and whether this prevents predators from adjusting their behavior over multiple interactions with prey (9, 10).

Controlled experiments in which human predators target continuously moving virtual prey have demonstrated that abrupt and unpredictable changes in direction reduce the accuracy of prey targeting (11, 12). However, it is unknown whether the survival advantage conferred by unpredictable motion also applies against nonhuman predators. Additionally, the escape responses of prey which are initially stationary are common in nature, as numerous prey taxa freeze once they have detected a potential threat or remain motionless to avoid detection by predators, before eventually fleeing only once a predator gets too close (1, 13–15). One way for stationary prey to be unpredictable is to vary the initial escape angle from one encounter to the next (16). This is a distinct tactic to the unpredictable movements made by prey which move continuously regardless of the presence of a predatory threat (6) or the abrupt turns made by some prey in anticipation of a predator’s attack (17). Although theoretical models predict that for a predator of a given speed, prey should select a single optimal escape trajectory which maximizes the distance from an approaching predator (18, 19), predators might anticipate the movements of prey which repeatedly escape at a fixed angle relative to their approach (20). Contrary to expectations based on a single optimal escape path, a wide range of prey species show a substantial degree of variability in their initial escape angles (16), including amphibians (21), crustaceans (22, 23), fish (24–27), insects (28, 29), mammals (30), and reptiles (31). Given that this variability is so widespread taxonomically, investigating whether it represents an antipredator strategy aimed at generating unpredictability could have major implications for our understanding of prey escape behavior (32, 33).

Many predator-prey interactions are typified by feedback between the attacker and the target (34), making it difficult to disentangle the effects of prey defenses on predators from the impact of predator behavior on prey using a purely observational approach. One way to determine the importance of prey defensive tactics is to present real predators with standardized virtual prey, whose movements and behavior can be precisely controlled and experimentally manipulated (35–39). However, previous experiments with virtual prey have used unresponsive prey items which do not react to predators, and do not allow the predator to capture prey and be rewarded, making it extremely challenging to study repeated interactions between predators and prey. These limitations can be overcome by using interactive robotic prey (40).

**Fig. 1.**
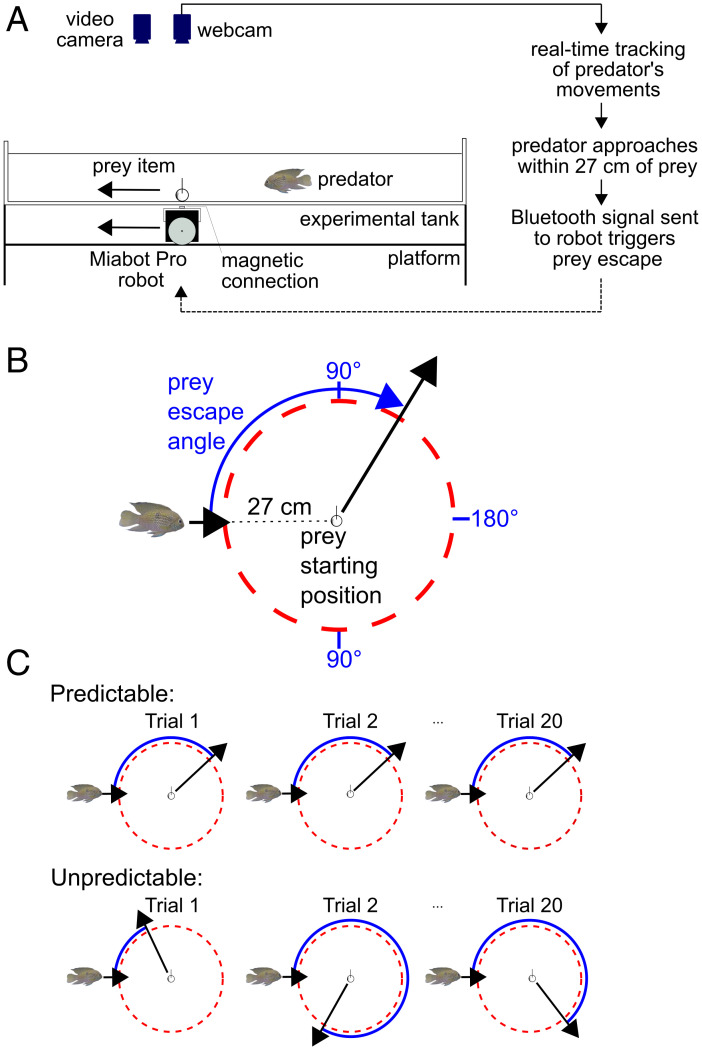
The robotic prey system. (*A*) Diagram (not to scale) showing a side view of the experimental system, with the Bluetooth-controlled robot situated on a platform underneath the experimental tank and the webcam used to monitor the predator’s movements suspended overhead. The prey’s movements are controlled by the robot via magnets, enabling the prey to escape from an attacking predator once the predator approaches within 27 cm of the prey’s starting position. See ref. 41 for a similar system designed for robotic predators attacking prey fish shoals. (*B*) Prey escape angles were defined relative to the predator’s approach direction. (*C*) In the predictable treatment, prey escaped at the same initial angle over successive trials (the escape angle varied between individual predators). In the unpredictable treatment, the prey’s initial escape angle varied randomly from trial to trial. While the experiment manipulated the prey’s initial escape angle, the prey’s subsequent escape trajectory was fixed as a straight-line path in both treatments.

To study the effect of unpredictability in prey escape on predators, we developed an experimental system [Fig. 1*A*; see also Swain et al. (41)], in which artificial robot-controlled prey were programmed to flee from blue acara cichlid (*Andinoacara pulcher*) predatory fish. Blue acaras are opportunistic predators with a broad diet but actively pursue highly evasive prey such as Trinidadian guppies (*Poecilia reticulata*) (42, 43). Prey initiated their escape response once the predator had approached within a threshold distance (Fig. 1*B*), mimicking the tendency of many prey to flee from a distant predator at submaximal speeds (14, 44). After an initial period in which groups of blue acaras were trained to attack the prey (the training period, *SI Appendix*, *SI Methods*), individual predators were repeatedly presented with prey which escaped either in predictable or unpredictable directions over 20 successive experimental trials (the test period). For individuals assigned to the predictable treatment (which acted as the control), prey escaped at the same angle relative to the predator’s approach from one trial to the next, whereas in the unpredictable treatment, prey were programmed to flee in random directions over successive trials (Fig. 1*C*). To successfully capture prey, pursuit predators must respond to changes in prey direction, which occur at the start of a chase (45–47). Across trials with predictable prey, the predators had the opportunity to gain reliable information about the prey’s likely escape direction, in contrast to the unpredictable treatment where the prey’s escape angle in previous trials was not a reliable indicator of its escape direction in future encounters. If unpredictable escape behavior is adaptive, increased uncertainty about the prey’s likely escape direction in the unpredictable treatment should reduce the performance of the predator in these trials, with slower speeds of approach (i.e., before the prey respond), longer times taken to capture prey, and/or greater kinematic costs resulting from higher speeds, increased acceleration, and more turning during the pursuit.

## Results

### Performance of the Robotic Prey System.

During the test period of the experiment, the predatory fish left the refuge in 532 out of a total of 540 trials and triggered the prey escape response in 524 of these trials (Movies S1–S3). There was no difference between the predictable and unpredictable treatments in the directional error of the escaping prey or in the prey’s reaction distance (*SI Appendix*, Table S1).

### Predator Behavior during the Approach Phase.

**Fig. 2.**
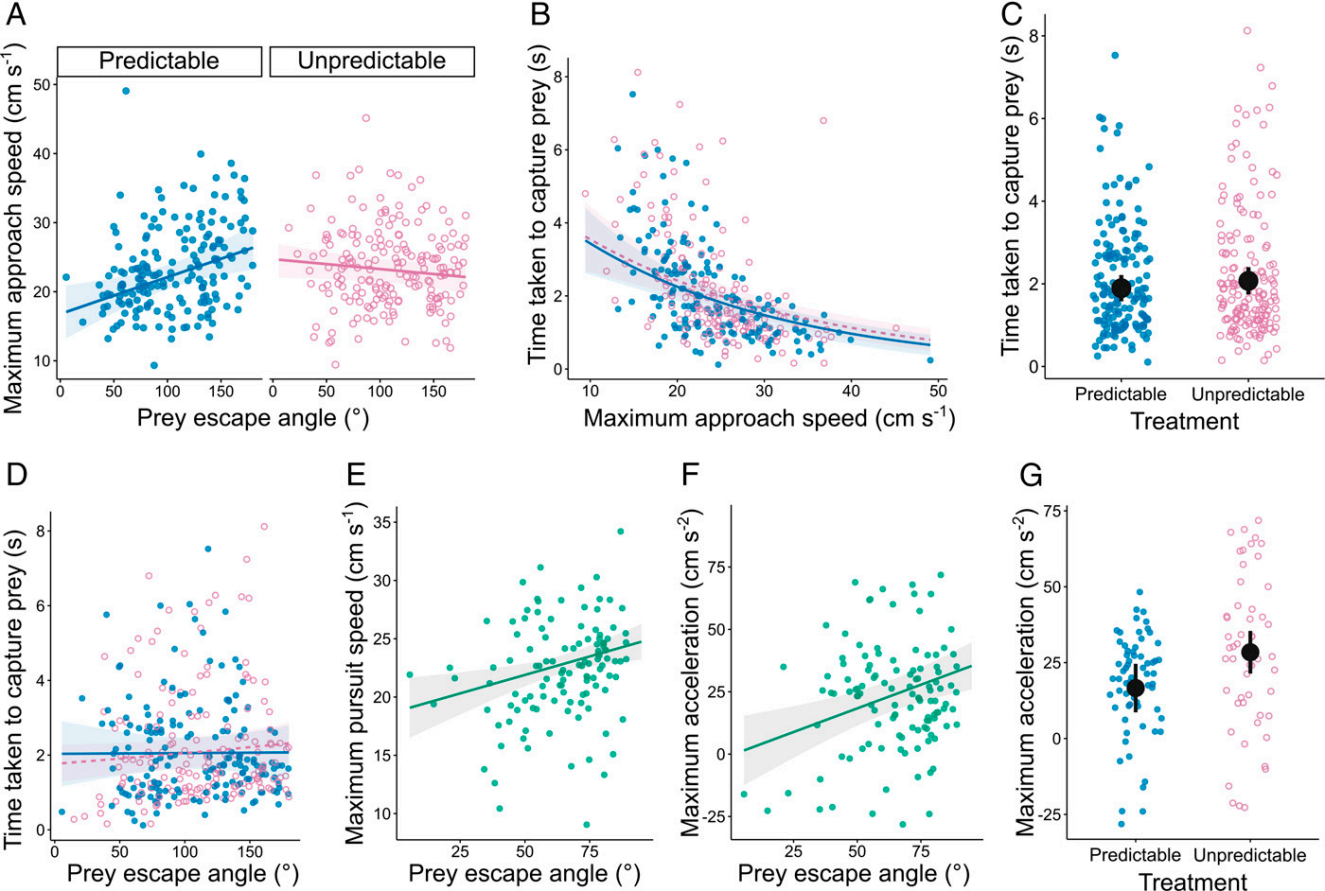
Behavior of the predator during the approach and pursuit phases. When approaching prey that escaped at a predictable angle, even before the prey began to respond, the predators adjusted their maximum speed by approaching prey expected to escape directly away from them (closer to 180°) more quickly (*A*). There was no effect of escape angle on the predators’ speed as they approached unpredictable prey, confirming that the predators in this treatment could not anticipate the prey's escape direction (*A*). Faster maximum approach speeds were strongly associated with reduced prey capture times once the prey began their escape (*B*), but there was no effect of treatment (*C*), or an interaction between treatment and approach speeds (*B*), on the time taken to capture prey. Prey escaping directly away from the predators took longer to capture (*D*), explaining why predictable prey expected to escape directly away from the predators were approached more rapidly than prey escaping at an acute (smaller) angle (*A*). In trials where prey escaped at an acute angle (<90°) (*E–G*), the predators pursued prey escaping at smaller angles more slowly than those escaping sideways (closer to 90°) (*E*), consistent with a reduced maximum acceleration (*F*). Predators pursuing unpredictable prey had greater maximum accelerations than those pursuing predictable prey (*G*). This at least partially compensated for not being able to adjust the maximum speed during the approach (*A*), which was related to the time taken to capture (*B*), but it does suggest a possible energetic cost of pursuing unpredictable prey. *A* and *E*–*G* show the fits from the top-supported models in Table 1 (*A*) and *SI Appendix*, Tables S4 (*E*) and S6 (*F* and *G*), with other explanatory variables held constant at their mean values. *B*–*D* show fits from models including an interaction between treatment and the predator’s maximum approach speed (*B* and *C*) and the prey’s escape angle (*D*). Separate lines of best fit for the predictable treatment (solid blue line) and unpredictable treatments (dashed pink line) are included in *B* and *D* to illustrate the absence of an effect of treatment on prey capture times (Table 2). Data points represent individual trials in the predictable (closed blue circles) and unpredictable (open pink circles) treatments. Shaded areas (*A*–*E*) and error bars (*C* and *G*) indicate the 95% confidence intervals surrounding the predicted values. Some prey escape angles were under 45°, as the programmed escape angles did not perfectly match the realized angles.

To investigate whether and how the predators adjusted their approach behavior in response to the prey escape strategy they encountered over successive trials, we compared a set of linear mixed-effects models (LMMs) predicting the predator’s maximum speed during the approach phase, i.e., the period before the prey escape response was triggered. Based on a comparison of values for Akaike’s information criterion corrected for small sample sizes (AICc), with a difference of two units or more indicating strong support for one model over another (48), the model including the interaction between treatment and the prey’s escape angle received the most support from the data, as shown by the improvement in model fit compared to both the baseline model and the model including the main effects of treatment and prey escape angle (Table 1 and *SI Appendix*, Table S2). In the predictable treatment, the predators reached higher maximum speeds when approaching prey which escaped directly away from them (Movie S1) compared to prey that escaped at an acute angle (Movie S2), but in the unpredictable treatment, there was no relationship between maximum approach speed and prey escape angle (Fig. 2*A*). The positive relationship observed in the predictable treatment was not explained by differences between individual predators in traits which could influence approach speeds, such as body size or a proxy for the predator’s motivation (*SI Appendix*, Table S3). There was no evidence for an effect of trial number on the predator’s maximum approach speed or an interaction between treatment and trial number (Table 1), suggesting that the prey’s predictability had no influence on how the predator’s maximum approach speed changed with further experience after the training period. The effect of trial number varied considerably between individual predators (*SI Appendix*, Fig. S1), as demonstrated by the large reduction in model fit when individual-level random slopes for trial number were removed from the top-supported model (ΔcAIC [change in the conditional Akaike information criterion between the two models]: 56.9).

**Table 1.** Results of LMMs explaining the predator’s maximum approach speed

Explanatory variables	Degrees of freedom	AICc	ΔAICc
Treatment × Prey escape angle	10	2222.3	0.00
Treatment × Trial number × Prey escape angle	14	2226.2	3.94
Trial number	8	2226.6	4.32
Baseline model (standard body length only)	7	2227.6	5.28
Treatment + Trial number	9	2228.2	5.96
Treatment × Trial number	10	2229.1	6.81
Prey escape angle	8	2229.5	7.28
Treatment + Prey escape angle	9	2231.2	8.91

To ensure the predator was motivated to attack and pursue prey, this analysis was limited to the 363 trials (featuring 25 individual predators) in which the predator approached the prey directly (i.e., the predator’s bearing to the prey was less than 45° when triggering the prey’s escape response) and subsequently captured the prey. All models included standard body length as an explanatory variable to control for individual differences in body size. All models included random intercepts for individual identity and training group and individual-level random slopes for the effect of trial number. A summary of the Treatment × Prey escape angle model, which received the most support from the data, is provided in *SI Appendix*, Table S2. For a given model, ΔAICc refers to difference between the model's AICc value and the AICc of the model receiving most support from the data.

### Effects on the Time Taken to Capture Prey.

The time taken by a predator to reach a target is a key factor determining the outcome of the predator-prey interaction (24). In our study, we therefore focused on the time taken to capture prey as the variable with greatest relevance to prey survival. The predator’s maximum approach speed had a large effect on the time taken to capture prey (the three models which included the predator’s maximum approach speed as an explanatory variable received more support from the data than the baseline model featuring only reaction distance; Table 2), with faster approaches resulting in reduced latencies to capture (Fig. 2*B*). Despite the strong negative relationship between approach speeds and capture times, and even though the fish in our study modified their approach speed based on the predictability of the prey’s escape direction (the Treatment × Prey escape angle interaction shown in Fig. 2*A*), there was no effect of predictability on prey capture times (Fig. 2*C* and Table 2). There was also evidence that increasing the prey’s escape angle increased the time taken to capture prey: the model including this main effect had the lowest AICc score by a margin of 0.65 units (Table 2; Fig. 2*D*; and *SI Appendix*, Table S2). This can explain why predators in the predictable prey treatment reached faster maximum speeds when they approached prey expected to escape directly away from them, as a higher maximum speed could compensate for the additional time needed to capture prey fleeing directly away. This apparent compensation for longer capture times can also help clarify why the effect of treatment and prey escape angle on the predator’s maximum approach speed (Fig. 2*A*) did not translate into an effect on the time taken to capture prey (Fig. 2*C*).

**Table 2.** Results of gamma GLMMs explaining the time taken to capture prey

Explanatory variables	Degrees of freedom	AICc	ΔAICc
Maximum predator approach speed + Prey escape angle	9	791.8	0.00
Maximum predator approach speed	8	792.4	0.65
Maximum predator approach speed × Prey escape angle	10	793.9	2.08
Maximum predator approach speed × Treatment	10	796.0	4.20
Prey escape angle	8	839.7	47.83
Baseline model (reaction distance only)	7	839.7	47.87
Treatment + Prey escape angle	9	841.8	49.94
Trial number	8	841.8	49.97
Treatment × Prey escape angle	10	843.5	51.65
Treatment + Trial number	9	843.9	52.08
Treatment × Trial number	10	844.9	53.10
Treatment × Trial number × Prey escape angle	14	850.7	58.85

As the prey were captured within less than 10 s in most trials (*SI Appendix*, Fig. S2), trials in which fish failed to capture prey within this time limit were excluded, because the predator was unlikely to be sufficiently motivated to pursue prey, resulting in 325 observations of 23 individual fish. All models included reaction distance as an explanatory variable to control for differences in the distance to the prey when the prey started to escape. All models included random intercepts for individual identity and training group and individual-level random slopes for the effect of trial number. A summary of the model receiving the most support from the data is provided in *SI Appendix*, Table S2.

### Predator Behavior during the Pursuit Phase.

The time to capture prey during the pursuit phase will depend on the speed, acceleration, and maneuverability of the predator (47, 49). To examine the consequences of the prey’s predictability and escape angle on the kinematics of the pursuit, we considered data from the 117 trials in both treatments in which prey escaped at an acute angle (<90°), where maneuverability would be most important. Predators which approached prey more rapidly reached higher maximum speeds during the subsequent pursuit (*SI Appendix*, Table S4), consistent with the faster capture times shown in Fig. 2*B*. After controlling for this effect, the maximum speed of the predator during the pursuit phase was faster if the prey escaped sideways (closer to 90°) rather than toward (closer to 45°) the predator (Fig. 2*E* and *SI Appendix*, Table S4). There was no effect of the prey’s predictability (treatment) on the maximum pursuit speed or of an interaction between treatment and the predator’s maximum approach speed, which would be expected if the effects of a rapid approach depended on the prey’s predictability (*SI Appendix*, Table S4). There was also no indication that the minimum speed of the predator during the first half of the pursuit was influenced by the predator’s maximum approach speed, the prey’s predictability, or the interaction between these two variables (*SI Appendix*, Table S5).

The faster maximum speed of predators attacking prey with higher escape angles, i.e., those closer to 90°, can be explained by greater maximum accelerations when pursuing these prey (Fig. 2*F* and *SI Appendix*, Table S6). There was also evidence for an effect of treatment on the maximum acceleration of the predator, where predators accelerated more when pursuing prey escaping in an unpredictable direction compared to predictable prey (Fig. 2*G* and *SI Appendix*, Table S6). Higher approach speeds were also associated with greater maximum deceleration during the first half of the pursuit (*SI Appendix*, Table S7), as would be expected following a quick approach. Additionally, there was some evidence that predators turned more rapidly when pursuing unpredictable prey, but this effect only became apparent when controlling for the positive effect of trial number on the predator’s maximum turning speed (*SI Appendix*, Table S8). There was no effect of maximum approach speed or treatment on how sharply the predator turned (minimum turn radius) while chasing prey (*SI Appendix*, Table S9).

## Discussion

The potential antipredator benefits of behaving unpredictably have long been recognized (6, 50, 51), yet it remained unclear how real predators respond to unpredictable escape tactics in their prey. By developing an experimental system in which robot-controlled prey fled from a blue acara cichlid predator, we manipulated whether each individual predator experienced predictable prey that always escaped at the same initial angle relative to the predator’s approach or whether this initial angle varied unpredictably from trial to trial. Our results demonstrate that the predators in our study adjust their approach behavior (their speed) when they are able to predict the direction in which prey will escape. Prey escaping directly away from the predators (closer to 180°) were approached more quickly even before the prey responded, which compensated for the longer time needed to capture such prey during the pursuit, compared to those escaping at an acute angle. If the predators attempted to minimize the time to capture prey, however, they should have approached prey expected to escape at acute angles just as rapidly, given that the benefit of a rapid approach applied equally across all prey escape angles. When examining the kinematics of the predator’s trajectory during the pursuit, even after controlling for the faster approach speed, the predators accelerated more and reached faster maximum speeds when pursuing prey fleeing at angles closer to 90° than 45°. These results suggest that with information on the prey’s likely escape direction, the predators were minimizing the costs of capturing prey and aimed to achieve an adequate, rather than minimal, time to capture.

In the unpredictable treatment, there was no relationship between an acara cichlid’s approach speed and the prey’s escape angle, confirming that the individuals in these trials could not anticipate the prey’s escape direction. Instead, compared to fish in the predictable treatment, these fish adopted intermediate approach speeds, which may represent a form of insurance aimed at buffering against the uncertainty caused by a lack of consistency in the prey’s escape direction (52). This behavioral adjustment is likely to represent a deliberate strategy, rather than a response to the confusion generated by the prey escaping in an unexpected direction. If predator confusion played a significant role, the predators would be expected to approach prey at much slower speeds, compared to those observed in the predictable treatment. Additionally, in trials where the prey escaped at an acute angle (<90°), there was also evidence that the predators accelerated more when pursuing unpredictable prey compared to predictable prey. This is consistent with these fish rapidly gaining ground on the prey after an approach where the predator did not have access to reliable information on the prey’s probable escape direction. Together, these behavioral adjustments enabled the predators facing unpredictable prey to nullify the expected benefits to prey of escaping unpredictably and thus attain comparable average prey capture times to individuals in the predictable treatment, even though they lacked information on the prey’s likely escape direction.

In our study, the variable most directly relevant to prey survival was the time taken to capture prey, as a longer delay increases the probability that the prey can find refuge or the predator gives up the pursuit (53–55). Since the approach speed of the predators in our study was a key determinant of the time taken to capture prey during the pursuit, one possible interpretation of our findings is that escaping unpredictably is advantageous to prey because it causes predators to approach at intermediate speeds which are suboptimal for prey capture. Crucially, however, we did not observe an effect of prey predictability on prey capture times, either as a main effect or as part of an interaction. This suggests that the behavioral counteradaptation shown by the predators in our study was sufficient to negate the potential benefits (to prey) of escaping in an unpredictable direction, bringing into question, at least in some circumstances, whether unpredictable escape behavior has an adaptive benefit to prey. Instead, the unpredictable behavior observed in real prey (21–31) may come about through biomechanical or sensory constraints on the angle of escape (32) or simply because a variety of escape directions are equally effective (56). Although we find no evidence that unpredictable escape tactics are directly advantageous to prey, this strategy could be beneficial in other contexts. When faced with prey escaping at an acute angle, the predators in our study accelerated more when pursuing unpredictable compared to predictable prey, which could result in the accumulation of energetic costs for predators over longer pursuits where prey repeatedly change their direction unpredictably (49, 57, 58). If there is a greater cost of preying upon unpredictable prey, this may result in predators switching to alternative targets (for prey in groups) or other prey types (59, 60).

A major advantage of the experimental system we developed lies in the ability to tightly control both the behavior of the robotic prey and the experience of individual predators. Nonetheless, as with any experimental study performed in the laboratory, it is also vital to identify the real-world contexts to which the findings are most likely relevant. Rather than precisely replicating a specific predator-prey system, and limiting the relevance of our findings to a specific case, our study was designed to test a widely held assumption about prey escape behavior by reproducing only a few key features of prey behavior. These included the tendency of numerous prey species to remain stationary before fleeing (1, 13–15) and the widely observed variability in the initial escape angles of real prey (21–31). Importantly, however, instead of being designed to mimic rapid fast-start escape responses which are executed within extremely close range of the predator (24), our study more closely resembles a situation where prey escape at a relatively low speeds, which tends to occur when the predator is some distance away (14, 44). Additionally, since blue acaras are opportunistic predators in their natural environment that actively pursue prey such as guppies (43, 61), the results of our study are most likely to be applicable to predator-prey interactions involving pursuit predators like blue acaras, which adjust their trajectory in response to fleeing prey (62, 63). For ambush or stalk-and-strike predators, which typically do not adjust their trajectories in the period immediately after striking (64, 65), unpredictable prey escape behavior may be more effective than in our study, suggesting fruitful avenues for follow-up research. Future studies should also consider testing the effectiveness of evasive responses which combine unpredictability in the prey’s initial escape direction with randomness in the subsequent escape path (7, 8). Our results highlight how the success of unpredictable prey escape tactics is likely to be contingent on the capacity of the predator to counteradapt. In doing so, we underscore the importance of considering predators as responsive participants within predator-prey interactions (66, 67), that are capable of flexibly adjusting their behavior depending on the prey’s tactics.

## Materials and Methods

### Experimental Subjects and Housing.

A total of 28 blue acara cichlids (*A. pulcher*), captively bred at the University of Bristol from stock supplied by the University of Exeter, were tested in the experiment (median standard body length = 6.2 cm, interquartile range = 1.95 cm). The first 16 fish were tested in November and December 2018, with an additional 12 fish tested in February and March 2019. Outside of the experimental period, fish were kept in glass tanks (width = 40 cm, length = 70.5 cm, height = 35.5 cm), with a daily 12:12 h dark:light cycle and water temperature maintained from 26 to 27 °C (±0.5 °C). During the experiment, groups of four fish of different sizes were kept in a holding zone located at one end of the experimental arena, enabling individuals to be identified. Throughout the experiment, fish were fed ad libitum on aquarium fish pellets (ZM Systems, Large Premium Granular) at the end of each day.

### Experimental Setup and Robotic Prey System.

The robotic prey system included a rectangular tank divided into an experimental arena and a holding zone (*SI Appendix*, Fig. S3*A*); a Bluetooth-controlled robot (MiaBot PRO BT v2, Merlin Systems Corp. Ltd.) on a wooden platform suspended below the experimental tank (Fig. 1*A*); an artificial prey item located within the experimental tank itself (*SI Appendix*, Fig. S3*B*); a webcam (Logitech C920 USB Pro) positioned above the tank, which was used to monitor the movement of the predator; and a Bluetooth-enabled laptop connected to the webcam via a USB (universal serial bus) cable. Detection of the moving predator was integrated with robot movement commands via a custom-built program (*SI Appendix*, Fig. S4). The prey’s movements were controlled by the robot through a connection between magnets embedded in the base of the prey item and in a plastic hood on top of the robot (*SI Appendix*, *SI Methods*). Trials were filmed using a camcorder (Panasonic SD 800; resolution = 1,920 × 1,080 pixels, frame rate = 25 frames per second) suspended 225 cm above the experimental tank.

### Training Period.

Before being trained to attack artificial prey, fish were tested individually for boldness by recording the time taken to leave the refuge in two separate trials conducted 2 days apart (data from these trials were not used in this study). As individuals in groups tend to behave more boldly than lone individuals (68), groups of fish were then progressively trained to approach and take food from the artificial prey item in a series of training trials (*SI Appendix*, *SI Methods*).

### Test Period.

After completing the training period, fish were tested individually in 20 successive experimental trials with either predictable or unpredictable prey (Fig. 1*C*). Prior to the start of each 10-min trial, fish were transferred to the central refuge and left to habituate for 3 min. After 3 min, the sliding door was opened, allowing fish to enter the experimental arena.

Experimental trials were conducted between 0900 and 1700 and took place in three 6-day blocks and one final 2-day block, with a 1-day gap occurring after each block. Individuals were allocated randomly to either the predictable or the unpredictable treatment, subject to the constraint that a group of fish from the same holding compartment was split equally between the two treatments, with the largest two fish in each group assigned to different treatments. Individuals were tested in a randomized order on each day. In the unpredictable treatment, escape angles were generated randomly in each trial, and in the predictable treatment, trials with the same individual were conducted with a single randomly generated escape angle. In both treatments, the robot was programmed to escape at a speed of 15.8 cm s^−1^, comparable to the range of escape speeds observed in Álvarez and Metcalfe (69), da Silva et al. (70), and Webb (44) when prey flee from a more distant predator. Prey escape angles were chosen from a uniform distribution from 45° to 315° (where 0° was defined as the predator’s approach angle).

### Video Analysis.

ToxTrac (version 2.84) was used to extract the position of the predator in each video frame up to 30 s before and after the prey escape response was triggered (71). Prey coordinates were extracted manually from each frame using a custom-built program written in Python (version 3.6.9). Multiple behavioral variables were also manually extracted from videos: the time taken for the predator to leave the refuge, relative to the door opening; the time taken to trigger the prey escape response, relative to the predator’s emergence from the refuge (a measure of the predator’s motivation to pursue prey); and the time taken to capture prey, defined as the time difference between the start of the prey’s escape response and the moment when the predator made physical contact with the prey.

All movement variables were calculated from the raw positional data using R version 3.5.1 (72). Predator and prey trajectories were combined to calculate the predator’s bearing to the prey, defined as the absolute angular difference between the predator’s heading and the straight line connecting the predator and the prey. Since spurious changes in heading might result from errors when tracking a stationary predator, heading angles were only calculated when the predator had moved a minimum distance of 0.5 cm between frames. Reaction distances were calculated as the distance between the predator and prey in the video frame immediately before the escape response was initiated. Kinematic variables based on the speed of the predator were obtained by smoothing raw speed values using a locally weighted regression (LOESS) procedure. The predator’s turning performance was assessed by calculating its maximum turn speed and minimum turn radius (73). Further details of the procedures used to obtain kinematic variables are described in *SI Appendix*, *SI Methods*.

### Statistical Analysis.

All statistical analyses were conducted in R version 3.5.1 (72). LMMs and GLMMs were used to explore the variables that impacted the approach and pursuit behavior of the predators. Throughout the analysis, the relative influence of explanatory variables was assessed using AICc values to compare the level of support from the data between models within a set of candidate models (*SI Appendix*, *SI Methods* for further details).

## Supplementary Material

Supplementary File

Supplementary File

Supplementary File

Supplementary File

Supplementary File

## Data Availability

All study data are included in the *SI Appendix* and Dataset S1.
