# Supplementary material for: Responsive robotic prey reveal how predators adapt to predictability in escape tactics
Source: Proc Natl Acad Sci U S A. 2022 Jun 3;119(23):e2117858119. doi: 10.1073/pnas.2117858119 (PMC9191677; doi:10.1073/pnas.2117858119)
Supplement: Supplementary File [file pnas.2117858119.sapp.pdf]

PNAS

Supplementary Information for
Responsive robotic prey reveal how predators adapt to predictability in escape tactics

Andrew W. Szopa-Comley[1]* and Christos C. Ioannou[1]

Corresponding author (*): Andrew W. Szopa-Comley
Email: a.szopa-comley@bristol.ac.uk

This PDF file includes:

Supplementary text: SI Methods
Figures S1 to S4
Tables S1 to S9
Legends for Movies S1 to S3
Legend for Dataset S1
SI References

Other supplementary materials for this manuscript include the following:

Movies S1 to S3
Dataset S1

Supplementary Information Text: SI Methods

Experimental set-up and robotic prey system. In both training and test trials in which the prey was programmed to respond to an approaching predator, the artificial prey item was placed in the same starting position within the experimental arena (**Fig. S3a**). By processing footage captured by the webcam, a custom-built program (**Fig. S4**) was used to trigger the prey's escape response by sending movement commands to the robot (Miabot Pro BT v2, Merlin Systems Corp. Ltd.) once the predator had approached within 27 cm of the prey's starting position. The robot consisted of two wheels set within either side of a 7.5 cm cube containing the electronic circuitry, batteries and separate motors for each wheel.

In all escape responses, the robot was programmed to move a total of 38.5 cm in a straight line from its starting position towards the periphery of the tank. The turning time of the robot was standardised across training and test trials by including a 0.3 second time delay within the program, enforcing a consistent time lag between the initial turn command being sent to the robot via Bluetooth and the subsequent movement command which directed the robot to escape in a straight line. Pilot trials filmed at a frame rate of 240 frames per second (GoPro Hero 5, resolution: 1280 x 720 pixels) indicated that the mean overall time delay between the predator moving within range and the robot moving was 0.7 seconds (n = 7, standard deviation: 0.08 seconds).

Training period. During the training period, groups of fish were progressively trained to approach and take food from the artificial prey item in a series of training trials with three sequential training stages. Training trials were conducted in the same groups that the fish were housed in. Groups progressed to the next training stage once a pre-specified criterion was reached, ensuring all groups were trained to a similar level. Before each training trial, groups of fish were transferred to the central refuge and left to habituate for 3 minutes. After 3 minutes, the sliding door was opened, allowing fish to enter the experimental arena. Training trials lasted 10 minutes. At each stage of the training process, groups were subjected to three training trials per day.

In the first stage of the training period, a static, baited prey item was positioned 25 cm from the entrance of the refuge, surrounded by a small amount of food (12 cichlid pellets). Successful trials were those in which at least one fish from the group consumed a pellet and progression to the next stage occurred after success in 6 consecutive training trials. Training trials in the second stage were identical to those in the first stage, but the prey item was placed at the centre of the experimental arena, in the same location as the test trials. In stage two (and stage three), successful trials were those where at least one fish from the group consumed the food attached to the artificial prey item. Success in 10 consecutive trials was required to progress to the third (final) stage of the training period. The baited, robot-controlled prey item was programmed to initiate an escape response when a fish approached within the set distance (27 cm) at a speed set at 7.9 cm s^{-1} (half the speed used in experimental trials). To ensure that the training process did not bias the response of fish towards either of the two experimental treatments, during the third training stage groups were exposed to prey which escaped consistently in the same direction, as well as prey which escaped at a random angle. Within each pair of consecutive training trials (i.e. the pair formed by the first and second training trials, third and fourth trials, fifth and sixth trials, etc.), groups of fish experienced one trial with consistently escaping prey and one trial with randomly escaping prey. Within a pair of training trials, the order of consistent and random trials within each pair of trials was randomised for each group of fish. This way of determining trial order was repeated within pairs of consecutive training trials, until the fish had reached the criterion for progression to the test period. The prey's escape angle during consistent trials was chosen separately for each group of fish. To prevent prey moving directly back towards an attacking predator, in both consistent and random trials, escape angles were chosen from a uniform distribution ranging from 45° to 315° (where 0° was defined as the approach angle of the predator; **Fig. S3a**).

Video analysis. As the prey's actual escape angle sometimes deviated from the programmed escape path, all statistical analyses were based on realised escape angles, calculated from the known start and end points of the prey's escape trajectory. Additionally, although the prey was

programmed to respond when the predator had approached within 27 cm, the actual prey reaction distance varied due to a combination of a short delay between the predator being detected and the initial movement of the prey and differences in predator approach speeds across trials. Reaction distances were therefore calculated as the distance between the predator and prey positions in the video frame immediately before the escape response was initiated.

LOESS procedure used to obtain smoothed speed values. In the period before the prey escape response was triggered, predators tended to accelerate in a straight line towards prey during their approach. As the only information available to the predator during the approach phase about the prey's subsequent escape was from its experience of previous trials, the predator's maximum approach speed in each trial was calculated to measure how prey predictability influenced predator behaviour. To obtain reliable estimates of maximum approach speeds and reduce noise, LOESS (locally weighted regression) with a smoother span of 0.1 was used to smooth raw speed values over time. To avoid generating a smoothed time series which includes negative values, which were sometimes produced when using LOESS to smooth data on the original scale, the raw speed values were log(x+1) transformed prior to smoothing. Smoothed speeds on the original scale were then obtained by applying the inverse of this transformation, resulting in positive smoothed speed values. The maximum predator approach speed was defined as the highest speed during the period of the approach phase in which the predator headed continuously towards the prey (i.e. the predator's bearing to the prey did not exceed 45°), and did not subsequently deviate from this overall direction by heading away from the target. The period up to 10 seconds before the prey started to escape was considered. Data from trials when predators triggered the prey escape response while approaching at a bearing of greater than 45° to the prey were not included in the analysis, as fish were unlikely to have been as motivated to attack in these trials.

Quantifying predator turning performance during the pursuit phase. To assess the turning performance of the predator, we calculated both maximum turn speed and minimum turn radius, to provide an indication of both how quickly and how sharply the predator turned when pursuing prey (1). Turn speed was defined as the change in the direction of the predator's heading in successive frames, and turn radius was calculated as the straight-line distance between the predator's position in frames i and $i+2$, divided by two times the sine of the change in the predator's heading θ between successive frames, i and $i+1$ (below, x and y indicate the x- and y-coordinates of the fish):

$$turn\ radius_i = \frac{\sqrt{(x_{i+2} - x_i)^2 + (y_{i+2} - y_i)^2}}{2\sin(\theta_i)}$$

Statistical analysis. Linear and generalised linear mixed effects models (LMMs/GLMMs) were used to explore the variables that impacted the approach and pursuit behaviour of the predators, using a model comparison approach. LMMs were fitted with the lme4 package and GLMMS were fitted with the glmmTMB package. To limit the number of candidate models being compared, simpler versions of three-way interaction models (models lacking the three-way interaction but retaining the constituent two-way interactions) were not included in the initial model comparison set (2, 3). These were only considered if the initial model comparison revealed that the three-way interaction had an important effect. Within model comparison sets, additional explanatory variables were also added to all models in order to account for potentially confounding effects such as the standard body length of the predator, reaction distance (used to control for differences in the distance to the prey when the prey escape response was triggered) and the predator's maximum approach speed (used to control for the expected effect of approach speed on pursuit speed or acceleration). An additional model featuring only these control variables was also included in each model comparison set to serve as the baseline for comparison with the other models. Where control variables were also of interest, a null model lacking any explanatory variables was also included.

Within each comparison set, all models shared the same random effects structure. To control for similar experiences during the training period, and to account for repeated measures of the same individuals, random intercepts for training group and individual identity were included in all models. Individual-level random slopes for the effect of trial number were also included in models with maximum predator approach speed and the time taken to capture prey as the response (4). The extent of inter-individual differences in the effect of trial number on maximum approach speed was also assessed by comparing the conditional AIC (cAIC) of an LMM with individual-level random slopes to an otherwise identical model lacking random slopes using the cAIC4 package in R (version: 0.9; 5). To aid model fitting, continuous explanatory variables were scaled prior to being included in the model, by subtracting the mean and dividing by the standard deviation. Model assumptions were checked by examining QQ-plots of the residuals, residuals vs. fitted values and the distribution of the conditional modes of the random intercepts. The DHARMa package (version: 0.2.7) was used to check model assumptions for GLMMs (6).

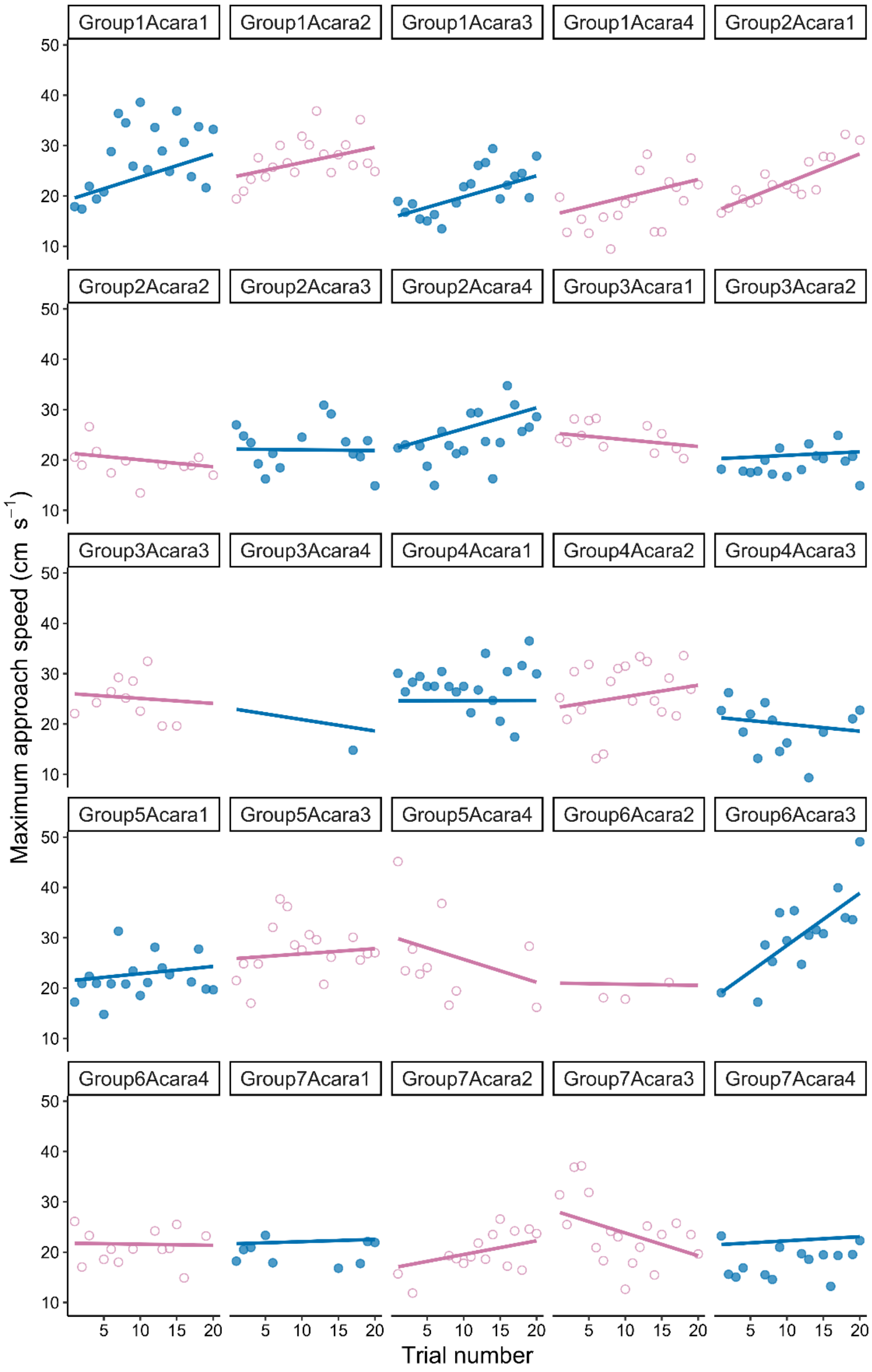

Fig. S1. The relationship between maximum approach speed and trial number, for each individual fish tested in the experiment. Colours indicate which treatment an individual was

assigned to (predictable: solid blue points; unpredictable: empty pink points). Individual predicted slopes were estimated using the *predict* function in the R package lme4.

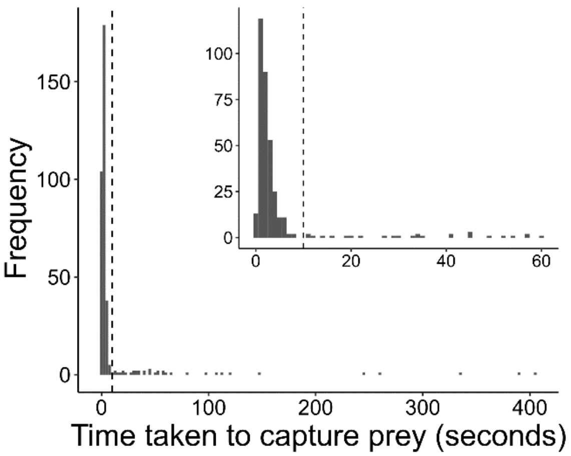

Fig. S2. The distribution of the time taken by the predator to capture prey. Data is shown for all 363 trials in which the fish left the refuge, and captured prey following a direct approach (i.e. trials in which the bearing of the predator to the prey was less than 45°, at the time when the prey escape response was triggered). The inset shows a magnified version of the same histogram, ranging from 0 to 60 seconds. In both the main histogram and the inset, the dashed vertical line indicates the 10 second threshold used to remove trials in which fish were insufficiently motivated to pursue the prey.

a)

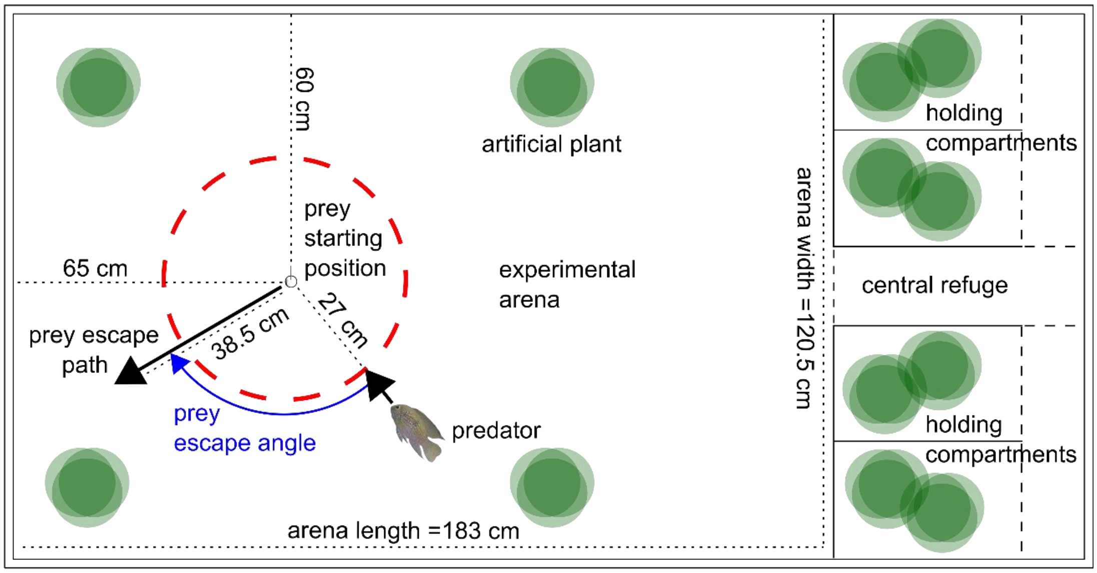

b)

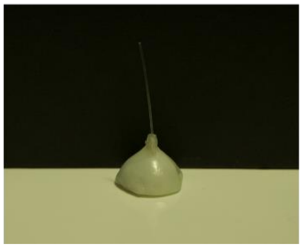

Fig. S3. Components of the robotic prey experimental system. (a) Scale diagram of the experimental arena, viewed from above. The red dashed line indicates the predator-prey distance (27 cm) at which the initially stationary robot-controlled prey item was programmed to escape from an approaching predator. Black arrows indicate the heading of the approaching predator and the escaping prey. The prey escape angle (relative to the approaching predator) is shown in blue. The experimental arena and fish holding zone were situated within a large rectangular aluminium tank (width = 127 cm, height = 36 cm, length = 238 cm). Within this external structure, an inner tank was constructed from white PVC walls (height = 35 cm, thickness = 0.8 cm) sealed to a white base made of compressed white foamed PVC (thickness = 0.2 cm) using aquarium sealant. This created a high contrast background enabling the movements of the predator to be tracked. The inner tank was divided into a large rectangular experimental arena, separated from a smaller holding zone by a white plastic divider, positioned at one end of the tank. The holding zone was further sub-divided into four compartments (width = 25.75 cm, length = 41.5 cm each), with two compartments positioned either side of a central refuge (width = 16 cm, length = 55.5 cm), which was covered by rigid plastic mesh. Each compartment contained a cylindrical tube and two artificial plants to provide cover for the fish. Small pebbles (< 0.5mm diameter) were also scattered across the floor of each compartment and the central refuge. Holding compartments were linked to the central refuge via a connecting corridor (width = 14 cm, length = 51.5 cm), which bordered the external wall of the inner tank. The holding compartments were separated from the central refuge and experimental arena by retractable doors (indicated by black dashed lines), enabling fish to be transferred from their holding compartments to the central refuge or released into the experimental arena without being caught in nets, thus minimising handling stress. The experimental arena also contained a water heater attached to the wall furthest from the holding zone, and four artificial plants positioned near the edges. Throughout the experiment,

the water depth in the experimental tank was kept at 15 cm, temperature was held constant at 27°C (+/- 0.5°C) and a 12h:12h light:dark cycle was maintained. Water was continuously filtered and circulated throughout the entire tank using two Eheim Classic 600 external canister filters, which took in water via inflow pipes positioned in the corners of the arena closest to the heater and discharged water back into the holding zone. (b) Photo of the artificial prey item used in the experiment, shown against a dark background. The artificial prey item was a small amount of food (an approximately 5 mm x 8 mm piece of defrosted fish) attached to a length of transparent monofilament fibre (thickness = 1 mm, length = 4 cm) protruding from a cone-shaped white plastic base (diameter = 2.5 cm, cone height = 1.8 cm).

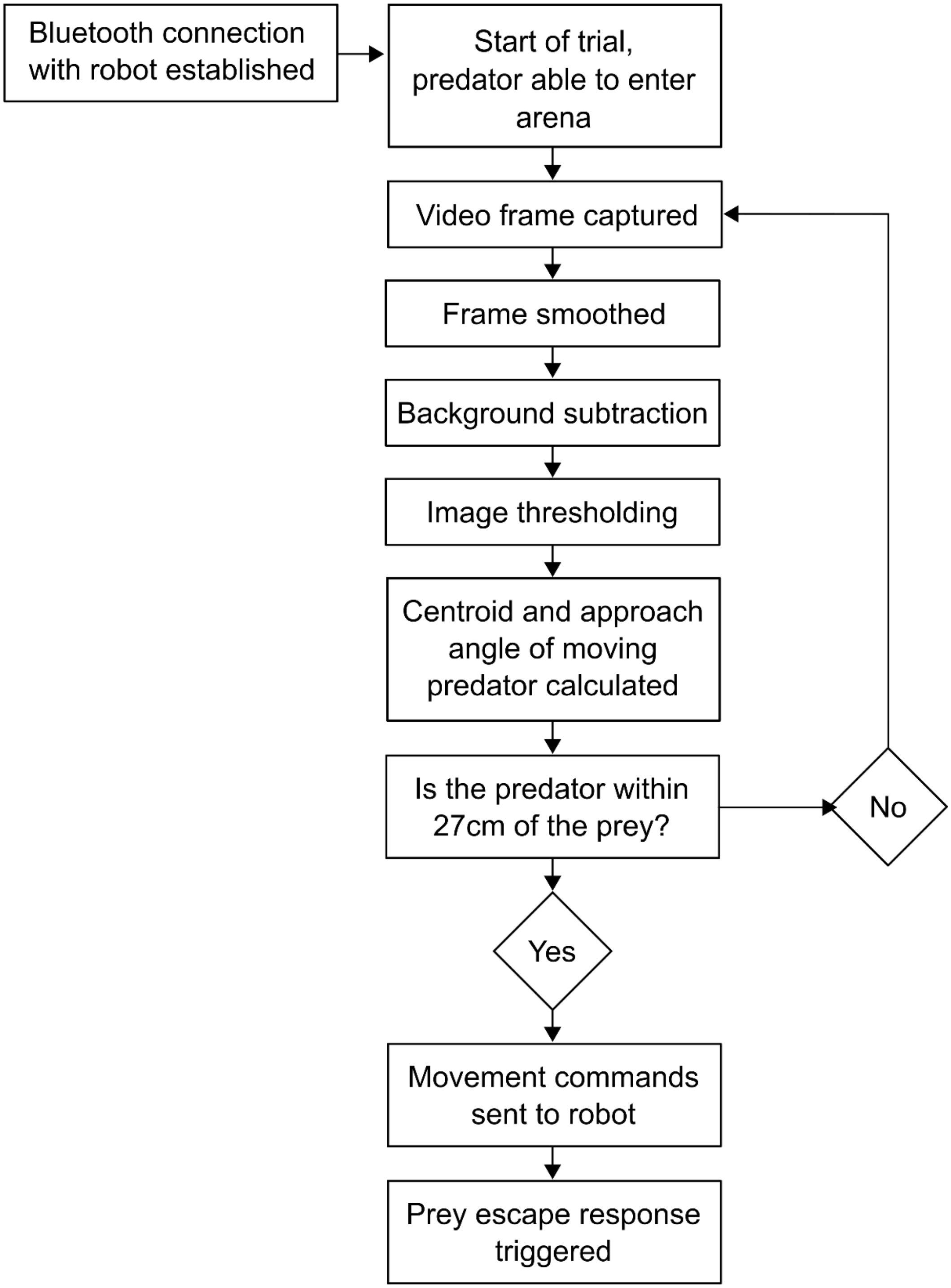

Fig S4. Schematic diagram showing steps involved in the program, written in python (version: 2.7.12) and utilising the OpenCV library (version: 3.1.0), which was used to control how prey respond to an approaching predator. Prior to an attack, the program continuously analysed video frames captured by the webcam positioned above the arena, monitoring any changes from one frame to the next which could indicate movement of the fish within the arena. As part of this process, each frame was first converted to grayscale and smoothed to filter out noise. Background subtraction was then used to calculate differences in pixel values between the current frame and a representation of the static background (i.e. the

unchanging aspects of the experimental arena). The background was estimated using a running average, in which motion during more recent frames was weighted more heavily. Thresholding was then applied to the resulting image to isolate areas of the current frame which differed substantially from the background. Additional size filtering also ensured that any regions of movement below a pre-specified size threshold were disregarded, to make certain that noise was ignored and that the sole region of detected motion corresponded to the predator. The program then used this information to calculate the centroid of the predator in each frame, until the predator approached within a pre-specified radius of the prey's starting position (27 cm in both training and test period trials). Video frame processing was halted at this point, and movement commands were sent to the robot, based on the predator's angle of approach and the programmed prey escape angle, in order to execute the prey's escape response.

Table S1. Analysing the performance of the robotic prey system. The table compares AICc scores for Gamma GLMs (generalised linear models) constructed to explain variation in the angular difference between expected and realised prey escape angles, and for LMMs (linear mixed-effects models) explaining variation in reaction distance. Throughout the experiment, the programmed (i.e. expected) prey escape angle did not always perfectly match the escape angle that was realised: the median angular difference between the expected and realised prey escape angle was 8.5° (inter-quartile range, IQR = 10.7°). The directional error of the robotic prey system did not differ between the predictable and unpredictable treatments and was not correlated with the expected prey escape angle (as demonstrated by the lack of improvement in fit for models featuring these explanatory variables, compared to the null model). Additionally, although the realised predator-prey distance at which the prey initiated its escape response (reaction distance) varied from trial to trial, there was no overall difference in reaction distance between the two treatments.

**Response variable**	**Explanatory variables**	**Degrees of freedom**	**AICc**	**ΔAICc**
Angular difference between expected and realised escape angles	Treatment	3	3756.5	0.00
	Null model (no explanatory variables)	2	3757.7	1.23
	Expected escape angle	3	3758.7	2.25
Reaction distance	Null model (no explanatory variables)	3	2281.2	0.00
	Treatment	4	2282.0	0.73

Table S2. Summaries of the statistical models receiving most support from the data, as determined by AICc-based model comparisons. Shown are coefficient estimates, their standard errors and the corresponding t- or z-values for each of the explanatory variables included as fixed effects within a model. Whereas t-values are reported for LMMs, z-values are reported for Gamma GLMMs. For each model, marginal R^2 values are also provided, as an overall measure of the variance explained by the fixed effects included in the model (7). n_{obs} refers to the number of trials within the data each model was fitted to. In all models, coefficient estimates for the effect of 'Treatment' are relative to the predictable treatment, which was set as the reference level.

Statistical model	Response variable	Explanatory variables	Estimate	S.E.	t value/z value	Marginal R^2
LMM (Table 1) n_{obs} = 363	Maximum predator approach speed	Standard Body Length	0.694	0.601	1.154	0.089
		Prey escape angle	2.136	0.713	2.997	
		Treatment	0.633	1.232	0.513	
		Prey escape angle x Treatment	-2.718	0.792	-3.431	
LMM (Table S3) n_{obs} = 179	Maximum predator approach speed	Prey escape angle	2.098	0.611	3.432	0.109
Gamma GLMM (Table 2) n_{obs} = 325	Time taken to capture prey	Reaction distance	0.197	0.028	7.139	0.357
		Maximum predator approach speed	-0.236	0.033	-7.399	
		Prey escape angle	0.053	0.033	1.664	
LMM (Table S4) n_{obs} = 117	Maximum predator pursuit speed	Reaction distance	1.501	0.438	3.428	0.148
		Maximum predator approach speed	1.326	0.446	2.975	
		Prey escape angle	2.542	0.858	2.963	
LMM (Table S6) n_{obs} = 117	Maximum predator acceleration during the pursuit	Reaction distance	4.873	2.038	2.392	0.133
		Maximum predator approach speed	-0.409	2.163	-0.189	
		Prey escape angle	15.202	4.443	3.422	
LMM (Table S6) n_{obs} = 117	Maximum predator acceleration during the pursuit	Reaction distance	4.201	2.116	1.985	0.131
		Maximum predator approach speed	-1.999	2.241	-0.892	
		Treatment	11.802	5.367	2.199	
LMM (Table S7) n_{obs} = 117	Maximum predator deceleration during the pursuit	Reaction distance	-1.690	1.594	-1.060	0.306
		Maximum predator approach speed	-10.519	1.664	-6.322	
		Trial number	3.024	1.285	2.354	

Table S3. Results of LMMs with the maximum approach speed of the predator during predictable treatment trials as the response variable, based on 179 observations of 12 individual fish. In the predictable treatment, individual predators were always exposed to prey escaping at the same angle. To test whether the positive relationship between the prey's escape angle and the predator's maximum approach speed (in predictable treatment trials; Fig. 1C) did not arise because of differences between individual fish, we examined which variables predicted the predator's approach speed. The model including prey escape angle received most support from the data, compared to models featuring standard body length, the approach speed of the predator in the first trial or the time taken to trigger the prey escape response (an indicator of the predator's motivation within a trial). This suggests that the relationship between maximum approach speed and prey escape angle observed in the predictable treatment (Fig. 1C) was unlikely to have arisen due to inter-individual variation in speed, body size or motivation. A summary of the model receiving most support from the data is provided in Table S2.

**Explanatory variables**	**Degrees of freedom**	**AICc**	**ΔAICc**
Prey escape angle	7	1082.0	0.00
Maximum approach speed in first trial	7	1087.0	4.97
Time taken to trigger the prey escape response	7	1087.7	5.67
Trial number	7	1088.0	6.03
Standard body length	7	1089.0	6.95
Null model (no explanatory variables)	6	1089.5	7.51

Table S4. Results of LMMs with the maximum speed of the predator over the course of the pursuit as the response variable, based on 117 observations of 19 individual fish in trials where prey escaped at an acute angle (< 90°). All models included reaction distance as an explanatory variable, to control for the effect of proximity to the prey at the point when the prey started to escape on changes in the predator's speed. Maximum predator approach speed was also included in all models except the baseline (reaction distance only) model, to control for the expected effect of approach speeds on the maximum pursuit speed. After controlling for the predator's approach speed, there was evidence for an effect of the prey's escape angle on the speed of the predator during the pursuit. This is shown by the relative improvement in fit (ΔAICc = 6.20) when comparing the model including fixed effects for both the predator's maximum approach speed and the prey's escape angle to the model including the predator's maximum approach speed (reaction distance was included in all models). A summary of this model, which received most support from the data, is provided in Table S2.

**Explanatory variables**	**Degrees of freedom**	**AICc**	**ΔAICc**
Maximum predator approach speed + Prey escape angle	7	668.3	0.00
Maximum predator approach speed	6	674.5	6.20
Maximum predator approach speed + Treatment	7	676.5	8.16
Maximum predator approach speed + Trial number	7	676.7	8.41
Maximum predator approach speed x Treatment	8	678.7	10.36
Maximum predator approach speed + Treatment + Trial number	8	678.7	10.43
Baseline model (Reaction distance only)	5	680.1	11.77
Maximum predator approach speed + Treatment x Trial number	9	681.0	12.73

Table S5. Results of LMMs with the minimum speed of the predator during the first half of the pursuit phase as the response variable, based on 117 observations of 19 individual fish in trials where prey escaped at an acute angle (< 90°). This first half of the pursuit was defined as period from when the prey started moving, until the time half-way between this start point and the moment the predator captured the prey. All models included reaction distance as a explanatory variable, to control for the effect of proximity to the prey at the point when the prey started to escape on changes in the predator's speed. Maximum predator approach speed was also included in all models except the baseline (reaction distance only) model, to control for the expected effect of approach speeds on the minimum pursuit speed.

**Explanatory variables**	**Degrees of freedom**	**AICc**	**ΔAICc**
Baseline model (Reaction distance only)	5	725.5	0.00
Maximum predator approach speed + Prey escape angle	7	726.7	1.18
Maximum predator approach speed	6	727.1	1.57
Maximum predator approach speed + Trial number	7	727.3	1.80
Maximum predator approach speed + Treatment	7	728.2	2.68
Maximum predator approach speed + Treatment + Trial number	8	728.8	3.24
Maximum predator approach speed x Treatment	8	730.5	4.98
Maximum predator approach speed + Treatment x Trial number	9	731.0	5.44

Table S6. Results of LMMs with the maximum acceleration of the predator throughout the pursuit as the response variable, based on 117 observations of 19 individual fish in trials where prey escaped at an acute angle (< 90°). All models included reaction distance as a explanatory variable, to control for the effect of proximity to the prey at the point when the prey started to escape on changes in the predator's speed. Maximum predator approach speed was also included in all models except the baseline (reaction distance only) model, to control for the expected effect of approach speeds on maximum acceleration in the pursuit. There was evidence for an effect of treatment on the maximum acceleration of the predator, as the model including maximum predator approach speed, treatment and reaction distance received greater support from the data than the model featuring only maximum predator approach speed and reaction distance (ΔAICc = 1.65). A summary of this model, and the model including maximum predator approach speed and prey escape angle as explanatory variables, is provided in Table S2.

**Explanatory variables**	**Degrees of freedom**	**AICc**	**ΔAICc**
Maximum predator approach speed + Prey escape angle	7	1030.9	0.00
Baseline model (Reaction distance only)	5	1037.7	6.77
Maximum predator approach speed + Treatment	7	1038.0	7.07
Maximum predator approach speed x Treatment	8	1038.7	7.77
Maximum predator approach speed + Treatment x Trial number	9	1038.8	7.89
Maximum predator approach speed	6	1039.6	8.72
Maximum predator approach speed + Treatment + Trial number	8	1040.3	9.37
Maximum predator approach speed + Trial number	7	1041.9	10.98

Table S7. Results of LMMs with the maximum deceleration of the predator over the first half of the pursuit as the response variable, based on 117 observations of 19 individual fish in trials where prey escaped at an acute angle (< 90°). This first half of the pursuit was defined as period from when the prey started moving, until the time half-way between this start point and the moment the predator captured the prey. All models included reaction distance as an explanatory variable, to control for the effect of proximity to the prey at the point when the prey started to escape on changes in the predator's speed. Maximum predator approach speed was also included in all models except the baseline (reaction distance only) model, to control for the expected effect of approach speeds on the maximum deceleration in the pursuit. Compared to a baseline model including maximum predator approach speed and reaction distance as explanatory variables, only the model featuring trial number, maximum predator approach speed and reaction distance represented a substantial improvement (a summary of this model is provided in Table S2). As the trials progressed, the fish decelerated more. The relatively large difference in AICc scores between the model including maximum predator approach speed and the baseline model indicated that the predator's maximum approach speed was strongly negatively correlated with its maximum deceleration during the pursuit phase, consistent with greater deceleration by rapidly approaching fish.

**Explanatory variables**	**Degrees of freedom**	**AICc**	**ΔAICc**
Maximum predator approach speed + Trial number	7	969.8	0.00
Maximum predator approach speed + Prey escape angle	7	971.3	1.44
Maximum predator approach speed + Treatment x Trial number	9	971.9	2.06
Maximum predator approach speed + Treatment + Trial number	8	972.1	2.26
Maximum predator approach speed	6	972.9	3.12
Maximum predator approach speed + Treatment	7	975.2	5.39
Maximum predator approach speed x Treatment	8	977.5	7.66
Baseline model (Reaction distance only)	5	1002.7	32.93

Table S8. Results of Gamma GLMMs with the maximum turning speed of the predator during the pursuit phase as the response variable, based on 116 observations of 19 individual fish in trials where prey escaped at an acute angle (< 90°). Only the model including trial number represented a substantial improvement over the null model, and as trials progressed, fish reached higher maximum turning speeds. The analysis was based on 116 observations, not 117, as the maximum turning speed could not be calculated in one of the trials where the predator always moved a distance of less than 0.5 cm between successive video frames (heading angles were only calculated when the predator had moved a distance greater than 0.5 cm between frames).

**Explanatory variables**	**Degrees of freedom**	**AICc**	**ΔAICc**
Treatment + Trial number	6	1416.6	0.00
Trial number	5	1418.0	1.47
Treatment x Trial number	7	1418.8	2.22
Maximum predator approach speed x Treatment	7	1425.6	9.02
Maximum predator approach speed	5	1425.7	9.11
Maximum predator approach speed + Treatment	6	1426.3	9.70
Prey escape angle	5	1427.2	10.67
Treatment	5	1427.3	10.72
Null model (no explanatory variables)	4	1427.3	10.73

Table S9. Results of LMMs with the minimum turn radius of the predator during the pursuit phase as the response variable, based on 115 observations of 19 individual fish in trials where prey escaped at an acute angle (< 90°). The analysis was based on 115 observations, not 116, as the minimum turn speed could not be calculated in an additional trial where the prey was captured within two video frames (a minimum of three frames were required to calculate minimum turn radii).

**Explanatory variables**	**Degrees of freedom**	**AICc**	**ΔAICc**
Trial number	5	502.7	0.00
Null model (no explanatory variables)	4	502.9	0.13
Prey escape angle	5	503.2	0.43
Treatment + Trial number	6	504.2	1.49
Treatment	5	504.6	1.83
Maximum predator approach speed	5	504.9	2.11
Treatment x Trial number	7	506.4	3.62
Maximum predator approach speed x Treatment	7	506.6	3.87
Maximum predator approach speed + Treatment	6	506.7	3.94

Movie S1 (separate file). Representative example of a predictable treatment trial, with the prey escaping directly away from the predator (close to 180°).

Movie S2 (separate file). Representative example of a predictable treatment trial, with the prey escaping directly at an acute angle from the predator (< 90°).

Movie S3 (separate file). Representative example of an unpredictable treatment trial, showing the predator accelerating towards the prey during the pursuit phase as the prey escapes.

Dataset S1 (separate file). Trial-level data on prey escape responses and predator behaviour.

SI References

1. S. A. Combes, D. E. Rundle, J. M. Iwasaki, J. D. Crall, Linking biomechanics and ecology through predator-prey interactions: flight performance of dragonflies and their prey. *J. Exp. Biol.* **215**, 903-913 (2012).
2. K. P. Burnham, D. R. Anderson. *Model selection and multimodel inference: a practical information-theoretic approach* (Springer Science & Business Media, New York, 2002).
3. X. A. Harrison, L. Donaldson, M. E. Correa-Cano, J. Evans, D. N. Fisher, C. E. D. Goodwin, B. S. Robinson, D. J. Hodgson, R. A. Inger, A brief introduction to mixed effects modelling and multi-model inference in ecology. *PeerJ*, **6**, e4794 (2018).
4. H. Schielzeth, W. Forstmeier, Conclusions beyond support: overconfident estimates in mixed models. *Behavioral Ecology*, **20**, 416-420 (2009).
5. B. Säfken, D. Rügamer, T. Kneib, S. Greven, Conditional model selection in mixed-effects models with cAIC4. https://arxiv.org/abs/1803.05664 (2018).
6. F. Hartig, DHARMa: Residual Diagnostics for Hierarchical (Multi-Level / Mixed) Regression Models. https://cran.r-project.org/web/packages/DHARMa/ (2020).
7. S. Nakagawa, H. Schielzeth, A general and simple method for obtaining R^2 from generalized linear mixed-effects models. *Methods. Ecol. Evol.* **4**, 133-142 (2012).
